# The essential molecular requirements for the transformation of normal cells into established cancer cells, with implications for a novel anti‐cancer agent

**DOI:** 10.1002/cnr2.1844

**Published:** 2023-06-06

**Authors:** Hilmar M. Warenius

**Affiliations:** ^1^ SYNTHERIX Ltd Clarendon House Cambridge UK

**Keywords:** apoptosis, carcinogenesis, cancer biology, drug design, mechanism of action, metabolism

## Abstract

**Background:**

Normal adult mammalian cells can respond to oncogenic somatic mutations by committing suicide through a well‐described, energy dependent process termed apoptosis. Cancer cells avoid oncogene promoted apoptosis. Oncogenic somatic mutations are widely acknowledged to be the cause of the relentless unconstrained cell proliferation which characterises cancer. But how does the normal cell with the very first oncogenic mutation survive to proliferate without undergoing apoptosis?

**New findings:**

The phenomena of malignant transformation by somatic mutation, apoptosis, aneuploidy, aerobic glycolysis and Cdk4 upregulation in carcinogenesis have each been extensively discussed separately in the literature but an overview explaining how they may be linked at the initiation of the cancer process has not previously proposed.

**Conclusion:**

A hypothesis is presented to explain how in addition to the initial oncogenic mutation, the expression of certain key normal genes is, counter‐intuitively, also required for successful malignant transformation from a normal cell to a cancer cell. The hypothesis provides an explanation for how the cyclic amphiphilic peptide HILR‐056, derived from peptides with homology to a hexapeptide in the C‐terminal region of Cdk4, kill cancer cells but not normal cell by necrosis rather than apoptosis.

## 1 INTRODUCTION

Cancer is widely accepted as being the result of oncogenic somatic mutations arising in normal cells, transforming them to undergo unconstrained division. The repeatedly dividing cells form a tumour which expands, invades local normal tissues and can spread from its site of origin via the lymphatic and/or blood systems to give rise to new metastatic sites of cancer.

The somatic mutation theory teaches that cancer begins with a genetic change in a single cell which passes it on to its progeny, thereby generating a clone of malignant cells.^1^ In addition to somatic mutation, first proposed by Boveri in 1914, non‐genomic properties of the cancer cell, some referred to as hallmarks, have also been identified as part of the cancer phenotype. As well as somatic mutations which drive oncogenesis, the cancer cell expresses a range of disparate characteristics including the avoidance of apoptosis, reliance on aerobic glycolysis and Cdk4 overexpression. These phenomena have previously, in general, been studied in isolation and mainly with regard to their activity within the phenotype of the already transformed cancer cell.

Here a hypothesis is presented to describe how these apparently separate phenomena may be connected to facilitate the initial oncogenic mutational event which triggers the emergence of a transformed cancer phenotype from within a normal diploid cell. This hypothesis also provides a potential mechanism to explain how a cyclic amphiphilic peptide anticancer agent with a warhead homologous to the Pro‐Arg‐Gly‐Pro‐Arg‐Pro sequence in a non‐kinase (NK) region on an external loop in the C′‐terminus of Cdk4, selectively kills cancer cells but not normal cells by depriving them of energy, in contrast to the classical anticancer drug mechanism of attacking their replication.

## 2 SOMATIC MUTATION

In‐vitro studies of malignant transformation of human bronchial epithelial cells have demonstrated that, in the presence of overexpression of Cdk4 and telomerase plus mutation of the p53 tumour suppressor gene, oncogenic expression of KRAS and MYC were sufficient to provide full, malignant transformation.^2^ Following the advent of whole human genome sequencing, however, Sjöblom^3^ showed that the number of mutational events occurring during the evolution of human tumours from a benign to a metastatic state was much larger than previously thought. An analysis of 13 023 genes in 11 breast and 11 colorectal cancers revealed that individual tumours accumulated an average of around 90 mutant genes. Only a subset of these contributed to the neoplastic process itself. Shortly afterwards, Greenman^4^ reported on among 1000 somatic mutations found in 274 Mb of DNA, corresponding to the coding exons of 518 protein kinase genes in 210 diverse human cancers. There was evidence for driver mutations in approximately 120 genes. A more recent analysis by Sondka et al.^5^ in the Catalogue of Somatic Mutations in Cancer (COSMIC) Cancer Gene Census (CGC) identified 719 cancer‐driving genes.

## 3 ANEUPLOIDY

Why increasing numbers of somatic mutations continue to be found in human cancer, with many more driver mutations than the minimum apparently required for a normal diploid cell to become transformed into a cancer cell in vitro, poses an interesting question. The development of genetic instability^6^ or a mutator phenotype^7^ once cancer has become established, have been proposed as potential explanations, but these develop late in the history of a cancer and are unlikely to be present during very early malignant transformation. The hypothesis described here provides an explanation for the development of aneuploidy, based on the avoidance of apoptosis from early in cancer development. The same mechanism is likely to happen with every new mutation occurring in the cancer cell as a result of genetic instability or a mutator phenotype once cancer is established. This could explain the increasing aneuploidy seen as cancers age and metastasize. The mechanism proposed here can only facilitate the progressive increase of aneuploidy because it tolerates further mutations. New driver mutations, however, may lead to different degrees of aggressivity and diversity of biological properties compatible with the considerable differences seen in cancer appearances (on histology etc.) and behaviour in the clinic.

## 4 AVOIDANCE OF APOPTOSIS

The concept that programmed cell death by apoptosis serves as a natural barrier to cancer development has been established by compelling functional studies.^8^ Apoptosis generally describes a process of energy‐dependent programmed cell death in response to a wide range of cellular conditions including: double‐strand breaks, DNA cross‐linking and telomere dysfunction. The types of somatic mutation which can cause cancer include gene amplification, point mutation, chromosomal rearrangements and epigenetic alterations such as DNA methylation. The somatic mutations underlying these phenomena are usually not significant enough for them to be responsible for inducing apoptosis on their own. It is replication stress during transformation of normal cells to cancer cells induced by somatic mutation that is generally understood to induce apoptosis rather than the mutation per se. This was initially demonstrated in early attempts to transform normal cells to cancer cells using an upregulated MYC oncogene, which could either result in apoptosis or increased cell division.^9^ Gerard Evan suggested that proliferation and apoptosis might be two opposing processes that Myc concurrently commandeered in cells. He suggested apoptosis was a potential ‘abort’ programme in case proliferation went wrong.^10^ Activated Ras proteins have also been described as causing apoptosis as well as aberrant cell division.^11^ During the initial process of oncogenic transformation, therefore, normal cells may undergo the energy‐dependent process of apoptosis rather than malignant transformation providing a general model for the consequences of oncogenic transformation by somatic mutation.

Reasons provided for escape from apoptosis by cancer cells are suggested to be increasing or decreasing expression of anti‐ or pro‐apoptotic genes, respectively or stabilising or de‐stabilising anti‐ or pro‐apoptotic proteins, respectively.^12^ Also because apoptosis is an energy dependent process, the relative availability of ATP can determine whether or not apoptosis occurs in a normal cell undergoing malignant transformation. High levels of ATP enable cells to undergo apoptosis, low levels of ATP shift cells away from apoptosis towards necrosis.^13^ Cancer cells navigate a narrow path between the Scylla of apoptosis and the Charybdis of necrosis. This understanding provides an explanation for the spontaneous cancer cell necrosis observed in many established human cancer cell lines even under optimal growth conditions.^14^ Levels of ATP which determine the choice between necrosis or apoptosis in normal cells have been shown to be influenced by the extent to which ATP is consumed by PARP activity,^15^ so that, for example, fibroblasts from PARP deficient (PARP^−/−^) mice are protected from necrotic death and ATP‐depletion but can still undergo apoptosis.

## 5 AEROBIC GLYCOLYSIS

**FIGURE 1:**
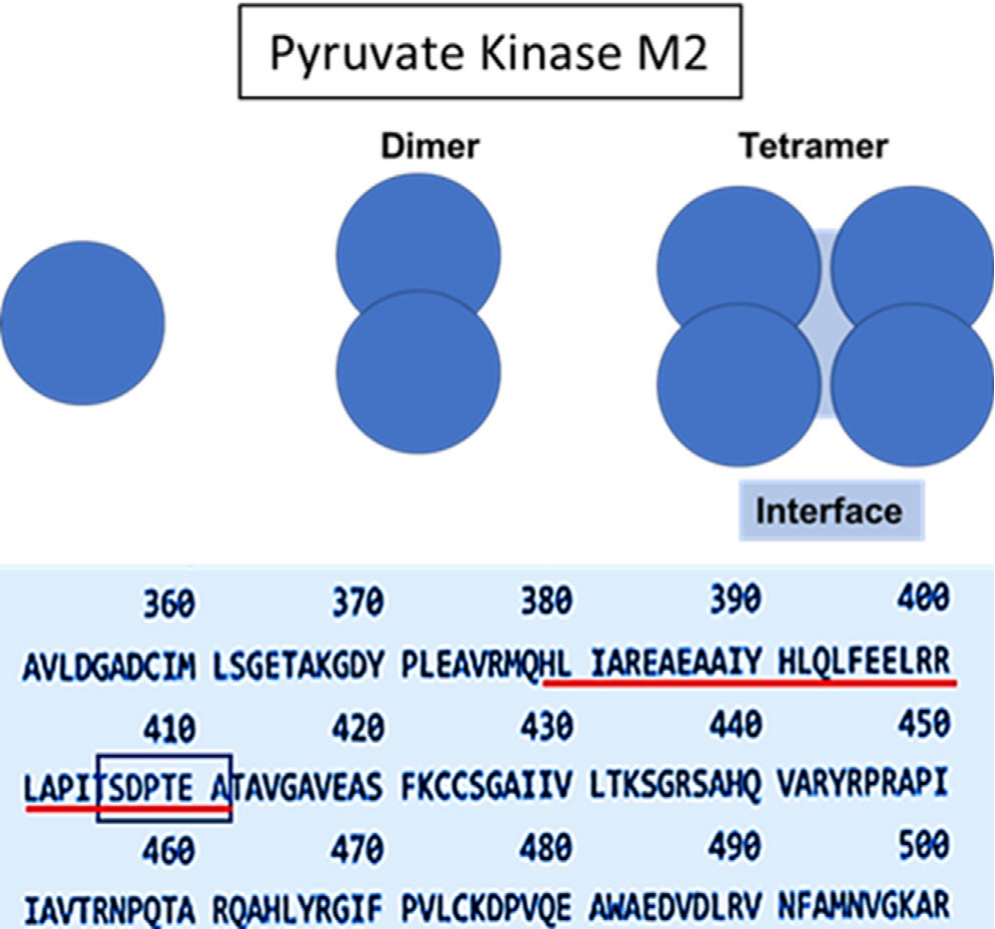
Schematic representation of PKM2 dimerization and tetramerization. The table shows the amino acid sequence of the peptide region contained within the tetramerisation interface (underlined in red). SDPTEA, the suggested putative target for PRGPRP, is at the right hand end of this sequence. Data extracted from the UniProt Blast (TrEMBL) amino‐acid sequence of human PKM2.

The level of the energy providing molecule ATP in cells is, however, primarily determined by carbohydrate metabolism. Nearly a century ago Otto Heinrich Warburg reported that carbohydrate metabolism in cancer cells, in contrast to normal cells, was restricted to the glycolytic pathway, avoiding much higher energy producing mitochondrial respiration.^16^ Although initially largely ignored, this cancer specific hallmark, subsequently termed aerobic glycolysis, has received progressively increasing interest^17^ It has been generally held that glycolysis in cancer cells is a consequence of anaerobic conditions in tumours that outgrow their blood supply and that this can provide an explanation for the phenomenon termed aerobic glycolysis^18^ an alternative theory holds that the type of carbohydrate metabolism found in tumours is a consequence of an exon 9/10 switch in the expression of the pyruvate kinase gene, resulting in the expression, of PKM2, rather than PKM1.^19^ The switch from exon 9 to exon to 10 of the pyruvate kinase gene codes for an amino acid sequence which differs from that in PKM1 between amino acid residues 378 and 411 (Figure 1, Underlined in red). This sequence is unique to cancer cells as opposed to normal cells in the adult and is located at the interface between two PKM2 dimers when they associate to form the active PKM2 tetramer responsible for generating ATP from phospho‐enol pyruvate.^20^

Hypoxia would not be expected to be present at the initial event causing malignant transformation of a normal cell. It is first seen in tumours that have reached a size of around 1–2 mm in diameter (corresponding to a volume of 1–8 mm^3^).^21^ If aerobic glycolysis is purely the result of hypoxia it would not be expected until clumps of cancer cells reach this volume. There is a sufficient body of evidence indicating that aerobic glycolysis occurs in the absence of hypoxia, however. For example, the Warburg effect can be found in early cancers in the absence of hypoxia and even normal adult tissues and premalignant tissues have been observed to manifest aerobic glycolysis. A study has reported that certain types of immune cells, such as activated T cells, exhibit a Warburg effect‐like phenotype during periods of increased activity.^22^

Also research examining the metabolic profile of human cervical cancer and normal cervical epithelium concluded aerobic glycolysis profiles clearly distinguished cervical precancerous lesions from normal cervical epithelium. Glucose consumption and lactate production was increased in cervical precancerous lesions and the expression of glycolytic enzymes LDHA, HK II and PKM2 also showed an increase in cervical precancerous lesions compared to normal cervical epithelium.^23^ Moreover, in rectal biopsies from 81 patients undergoing screening colonoscopy, normal appearing premalignant colorectal mucosal fields exhibit early Warburg‐like metabolic changes with upregulation of *HIF1α GLUT1 PKM2 and LDH* as well as changes in mitochondrial function.^24^

Overall, these and other studies imply that the Warburg effect is a common metabolic adaptation found in cancer and premalignant cells and may occur in the absence of hypoxia. In addition, hypoxia is not necessarily found in normal tissues where malignancy is typically known to arise. For example, detectable HIF‐1α is absent in normal ductal breast cells and hyperplastic lesions but elevated in breast cancers.^25^

There appears to be an underlying assumption that where aerobic glycolysis occurs in cancer, normal cells first become transformed into cancer cells and then these newly transformed cells “decide” in some way to adopt the aerobic glycolysis phenotype. It is difficult to understand why, normal cells transforming into cancer cells, should suddenly spontaneously adopt aerobic glycolysis. An alternative explanation, elaborated below, could be that the optimal conditions for the great majority of cancers to arise by malignant transformation are already present in cells in some regions of normal tissues which have already undergone the switch from PKM1 to PKM2 expression.

## 6 CYCLIN DEPENDENT KINASE 4

Upregulation of Cdk4 is one of the five phenotypic changes cited by Sato et al. as being minimal requirements for full malignant transformation. Human bronchial epithelial cells immortalised by overexpressing Cdk4 and human telomerase reverse transcriptase (hTERT) were employed as the starting point for their stepwise transformation process. These cells emulated two of the earliest and almost universal events in lung cancer pathogenesis: abrogation of the p16/Rb cell‐cycle checkpoint pathway and bypass of replicative senescence.^2^

Cdk4 classically acts as a kinase phosphorylating the Rb protein and releasing E2F when it is activated by being conjoined with cyclin D1 to form a holoenzyme^26^ Experiments in mice in which the wild‐type CDK4 gene on its own has been knocked down leaving normal Cyclin D activity, have, however, shown that without Cdk4 the induction of cancer by chemical carcinogenesis with 7,12‐dimethyl‐benz[a]anthracene applied to mouse skin, followed by the tumour promoter 12‐O‐tetradecanoylphorbol‐13‐acetate (TPA) was severely blocked. This was despite of the continuing presence of Cdk2 and Cdk6.^27^ Importantly Cdk4 knockdown prevented carcinogenesis in mouse skin but did not affect normal keratinocyte proliferation.

Also in the case of malignant transformation by oncogenes, Miliani de Marval et al. reported that lack of CDK4 expression due to gene knockdown in K5Myc transgenic mice resulted in the complete inhibition of tumour development, induced by deregulated Myc, suggesting that CDK4 is a critical mediator of tumour formation^28^ Furthermore, transformation in response to Ras activation with dominant‐negative (DN) p53 expression or in an Ink4a/Arf‐null background does not occur in CDK4‐null mouse embryonic fibroblasts, providing a further indication that Cdk4 is essential for immortalisation.^29^ In addition, CDK4 knockdown inhibits the onset and incidence of mammary carcinoma in MMTV‐Neu‐Cdk4−/− mice.^30^

There is thus a body of evidence which indicates that normal wild‐type Cdk4 alone (without cyclin D1) plays an important role in the initial stages of carcinogenesis. The contribution of my laboratory to this story has been the discovery of a previously unidentified site in a non‐kinase region of the C‐terminal portion of wild‐type Cdk4 which could provide an explanation for why Cdk4 can have a function which promotes malignant transformation without its cyclin D1 partner.

## 7 THE C‐TERMINAL REGION OF CDK4

During investigation of co‐expression of Cdk4 and Cdk1 which were found in a wide range of human cancers,^31^ human A 2780 ovarian cancer cells were transfected with wild‐type CDK4.^32^ There was a consequent elevation in Cdk4 expression accompanied by a similar rise in Cdk1 expression. Interestingly, however, there was not any increase in pRb phosphorylation. This indicated the possibility of a non‐kinase activity of Cdk4 in addition to its classical kinase role as a holoenzyme when combined with cyclin D1. Following a detailed study of what was known at the time about the structure of Cdk4 by the author, a site for a potentially functional non‐kinase activity unique to Cdk4 was sought by comparing the C‐terminal regions of Cdk4, Cdk2 and Cdk6 in collaboration with professors Jeremy Kilburn, Jonathan Essex and Dr Richard Maurer at the University of Southampton.

**FIGURE 2:**
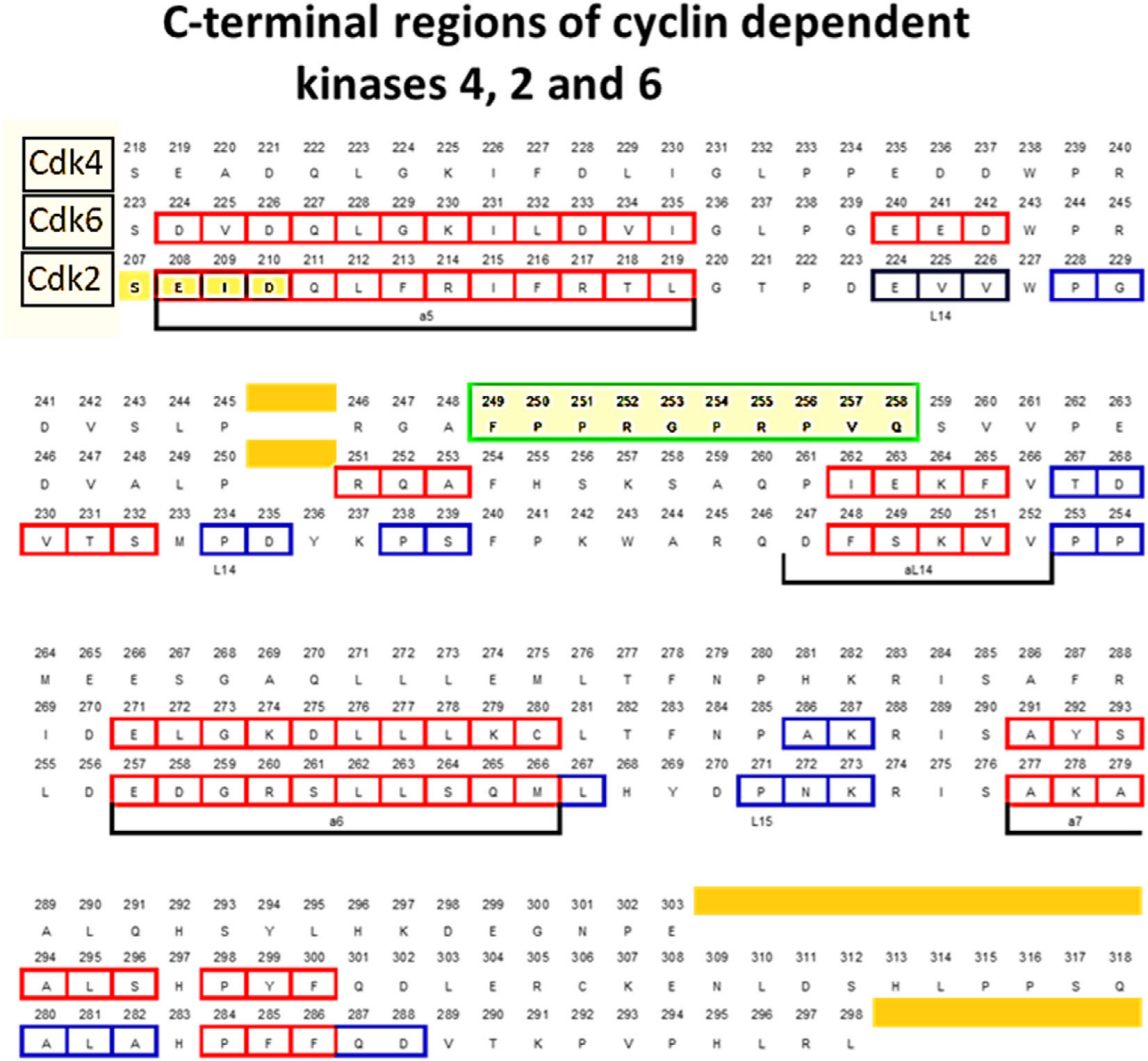
Comparison of the C′‐terminal regions of Cdk4, Cdk6 and Cdk2. The NKR region containing the central PRGPRP hexapeptide sequence is outlined in green with a yellow background. Red boxes how regions of homology. Blue boxes show regions of partial homology.

Figure 2 was constructed from the data collected in this earlier study^32^ but not previously published in this form. It clarifies the potential relevance of the NK region of Cdk4, which, in the light of the present hypothesis, assumes a new importance. Several homologous and partially homologous regions for Cdk2 and Cdk6 can be identified (red and blue boxes) but there are surprisingly none for this region of Cdk4.

Additionally, there is a unique decapeptide region in Cdk4, between amino‐acid residues 249 and 258 which has no similar region in Cdk6 or Cdk2. This has now been termed the non‐kinase (NK) region of Cdk4. This region though partially hydrophobic is displayed as an external loop on the Cdk4 peptide and carries the hexapeptide sequence: Pro‐Arg‐Gly‐Pro‐Arg‐Pro (PRGPRP). There have been a number of structural studies of Cdk4 but these have concerned models of its structure in combination with cyclin D, p16, Cdk37 etc. and/or predominantly focussed on the kinase region.

The comparison of the C′‐terminal regions of Cdk4, Cdk2 and Cdk6 has only been reported by Warenius et al.^32^ Images in the literature of 3D interactions of Cdk4 with other binding partners show the C‐terminal region and external loop containing PRGPRP are free and not involved in interactions of the known binding partners of Cdk4.^33^

The isolated PRGPRP peptide from the NK region of the cdk4 protein has been synthesised and placed in cyclic amphiphilic cassettes to allow internal access to cancer cells in tissue culture.^32^ In one cassette (THR53) it caused partial lowering of ATP accompanied by cellular necrosis. In a second cassette (HILR‐025) it produced a significant fall in the activity of LDH, a downstream indicator of PKM2 activity.

## 8 PYRUVATE KINASE M2

PKM2 regulates the rate‐limiting step of glycolysis that shifts glucose metabolism from the normal respiratory chain to lactate production in tumour cells. In its tetrameric form, pyruvate kinase M2 (PKM2) functions in glycolysis by transferring a phosphate from phosphenolpyruvate (PEP) to ADP to form pyruvate. In cancer cells dimeric PKM2 becomes abundant^34^ PKM2 as a protein kinase contributes to tumorigenesis^35^ and can be regulated by phosphorylation (for review see Reference 36). The dimeric form of PKM2 not only passively avoids participating in glycolysis but using PEP as a phosphate donor, catalyses tyrosine phosphorylation of STAT3.^37^

Several upstream molecules are known to modulate PKM2. Phosphorylation can occur on a number of amino acids. For example, Ser37 can be activated by ERK 1/2 promoting nuclear translocation.^38^ Tyr105 is often phosphorylated in tumours by fibroblast growth factor.^39^ Oncogenic phosphorylation of Tyr105 can impair tetramerisation by disrupting binding of FBP. Phosphorylation of Tyr105 can also induce cancer‐like stem cells and promotes breast cancer cell proliferation.^40^ Phosphorylation of Thr328 by HSP90 is critical to maintaining PKM2 stability and potentiating glycolysis.^41^ AKT phosphorylates Ser202 of pKM2, contributing to its nuclear import in response to insulin like growth factor.^42^ Phosphorylated PKM2 easily dimerises. EGFR promotes PKM2 transfer into the nucleus where it binds to phosphorylated‐Tyr333 of β‐catenin leading to expression of cyclin D.^43^ Nuclear PKM2 binds to and activates STAT5a which in turn upregulates cyclin D.^44^ Phosphorylation of PKM2 at Thr454 facilitates the transcriptional co‐activation of HIF‐1α and β‐catenin.^45^ PKM2 functions as a potential oncogene and is a crucial target of miR‐148a and miR‐326 in thyroid tumorigenesis.^46^ Also downregulation of PKM2 expression by miRNAs suppresses thyroid cancer. It has, however, conversely been shown that siRNA suppression of PKM2 expression decreased viability and caused apoptosis in multiple cancer cell lines but less so in normal fibroblasts or endothelial cells. A possible explanation for the above paradoxical findings is that knockdown of the whole of PKM2 would be expected to influence phenomena due to dimeric PKM2 alone as well as those due to tetramerization. Moreover, the above findings come from experiments on well‐established cell lines long after the initial transformative events resulting from early somatic mutations in normal cells that first created these established cell lines. A hypothesis is presented here concerning the events early in the transformation of normal cells to cancer cells which may potentially influence the tetramerisation process.

In contrast to the complex picture of modulation and activation of the PKM2 dimer, less information is available about the control of PKM2 tetramerization. PKM2 activators such as fructose‐1‐6‐biphosphate (FBP) promote allosteric changes which influence tetramer formation and suppress apoptosis which is abrogated by site directed mutation of PKM2 Cys‐423 within the tetramerization zone.^47^ A more detailed site is yet undiscovered. The single amino acid serine has also been shown to influence tetramerization.^48^

## 9 DIRECTLY INHIBITING PKM2

An early candidate for PKM2 inhibition was Shikonin, a naphthoquinone derived from the root of the Chinese plant Lithospermum erythrorhizon which has been reported as inducing death in a number of different cancer cell lines accompanied in most circumstances by apoptosis. Its precise mechanism is unclear but can involve pleiotropic interactions with signalling factors such as NF‐κB, PI3K/Akt/MAPKs, Akt/mTOR, TGF‐β, GSK3β, TLR4/Akt signalling pathways, NLRP3 inflammasome, reactive oxygen stress, Bax/Bcl‐2, etc.^49^

In addition to Shikonin, a number of more recent PKM2 inhibitors have been described (reviewed by Rathod et al.^50^). These include direct inhibitors such as compound 1, an irreversible inhibitor which has antiproliferative activity against several in vitro cancer cell lines at concentrations of 1–2 μM and impairs the growth of tumours in nude mice without acute toxicity. Compound 1 promotes apoptosis and binds via a terminal acetylene group to Cys326 near the PEP binding site and also to and Cys 317. Both of these residues lie outside the tetramerization site. Another compound is a thiazolidinedione which binds to the allosteric site of PKM2, releasing FBP. A similar allosteric site is not present on PKM1. Apoptosis is the mode of cell death that results from the PKM2 inhibition described above. Counterintuitively the isolated peptide homologous to PRGPRP in the NK region of Cdk4, causes death by necrosis rather than apoptosis. In contrast to PRGPRP, none of the inhibitors of PKM2 apart from thiazolidinedione described above, target the tetramerization region.

## 10 A POTENTIAL TARGET FOR PRGPRP

In an attempt to identify a potential target for PRGPRP that might explain its activity of partial depletion of ATP in cancer cells, a search of amino acid sequences of hexokinase, phosphofructokinase and pyruvate kinase was made. This revealed a potential binding site which mirrored PRGPRP by carrying two cationic acidic sites at similar distances apart within a hydrophobic site as the guanidium groups of PRGPRP.

**FIGURE 3:**
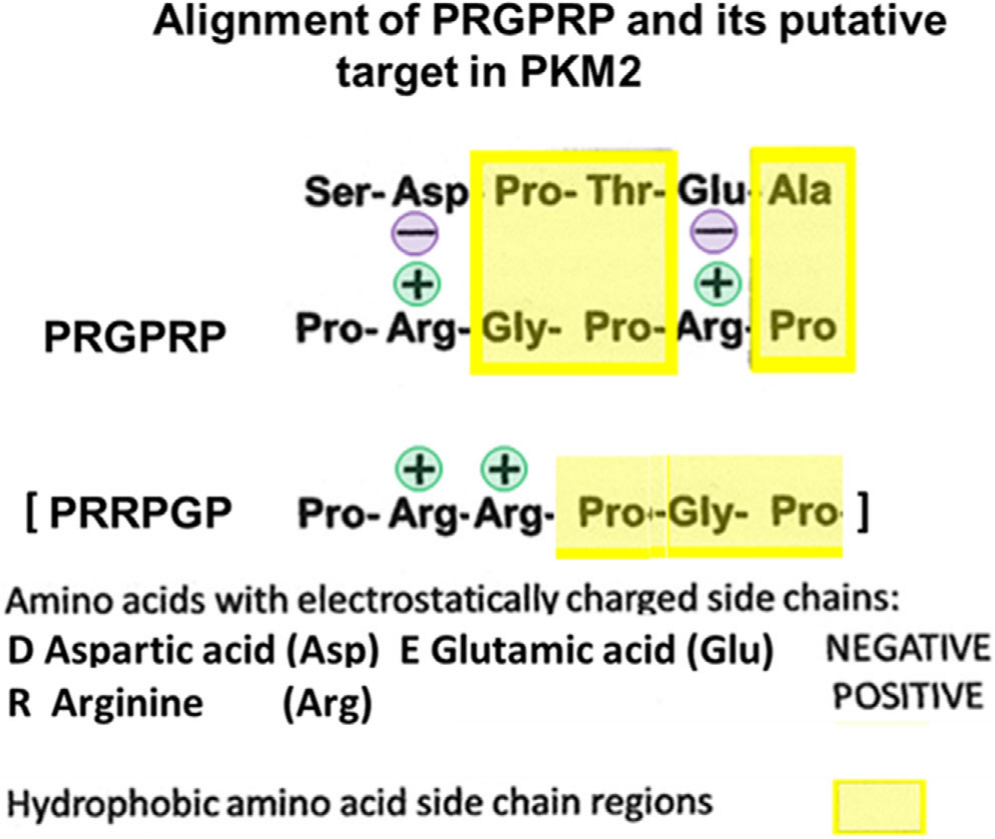
A schematic illustration of how the PRGPRP amino acid sequence from the NK region of Cdk4 could potentially align with the SDPTEA amino‐acid sequence at the right hand end of the interface of tetramerised PKM2 because of mutual attraction of the anionic guanidines to cationic aspartic and glutamic acids. PRRPGP would not align in this way.

The SDPTEA sequence at residues 406 to 411 lies in the interface between PKM2 dimers which tetramerize to produce active PKM2, containes two cationic amino acids in a non‐polar nest with a similar separation to the anionic arginines of PRGPRP (Figure 3). Strikingly the PRRPGP amino‐acid sequence, which had earlier been shown not to have the same necrosis inducing properties as PRGPRP^32^ despite the same amino‐acid constitution is clearly not a likely matching partner for SDPTEA.

It is proposed that the Cdk4 non‐kinase region we discovered may interact with PKM2 to limit ATP production and prevent the apoptosis in normal cells that would be expected to be the consequence of a random somatic mutation which promoted oncogenesis.

A new specifically designed assay to test the hypothesis that Cdk4 interacts with and modulates PKM2 activity is being designed to compare wild‐type Cdk4 with Cdk4 in which site directed mutations at positions 253 and 255 will provide the inactive sequence PRRPGP rather than PRGPRP. In addition computer simulations of potential docking of PRGPRP and SDPTEA are being undertaken.

## 11 AN ONCOGENE/NORMAL GENE INTERACTION HYPOTHESIS OF MALIGNANT TRANSFORMATION

Based on the previous literature discussed above and recent experimental findings I propose an hypothesis to explain how normal cells can avoid apoptosis at the transforming moment that the first oncogenic mutation occurs in a normal cell if it is expressing the PKM2 phenotype. Counterintuitively the functions of two normal cellular genes may be required to interact with the abnormal oncogene produced by a somatic mutation in order to transform normal cells into cancer cells. The potential importance of normal genes in malignant transformation was initially proposed 20 years ago (Warenius, 2002).^51^

The oncogenic mutation in a normal cell which initiates cancer upregulates Cdk4. In addition to promoting transit from the G1 phase of the cell cycle to S‐phase by the classical pathway of Cdk4/cyclin D1 phosphorylation of the Rb protein and release of E2F, the NK region of Cdk4 downregulates PKM2 so that there is not sufficient ATP for apoptosis. A transformed cancer cell with a PKM2 phenotype committed to aerobic glycolysis bearing a somatic oncogenic mutation is thus born. A similar process happens with the next mutation. With each iteration of a new mutation occurring in a genetically unstable cell, there is increased accumulation of mutations leading to aneuploidy. The process is likely to continue relentlessly and to progress far past the minimum number of driver mutations required for full malignant transformation.

**FIGURE 4:**
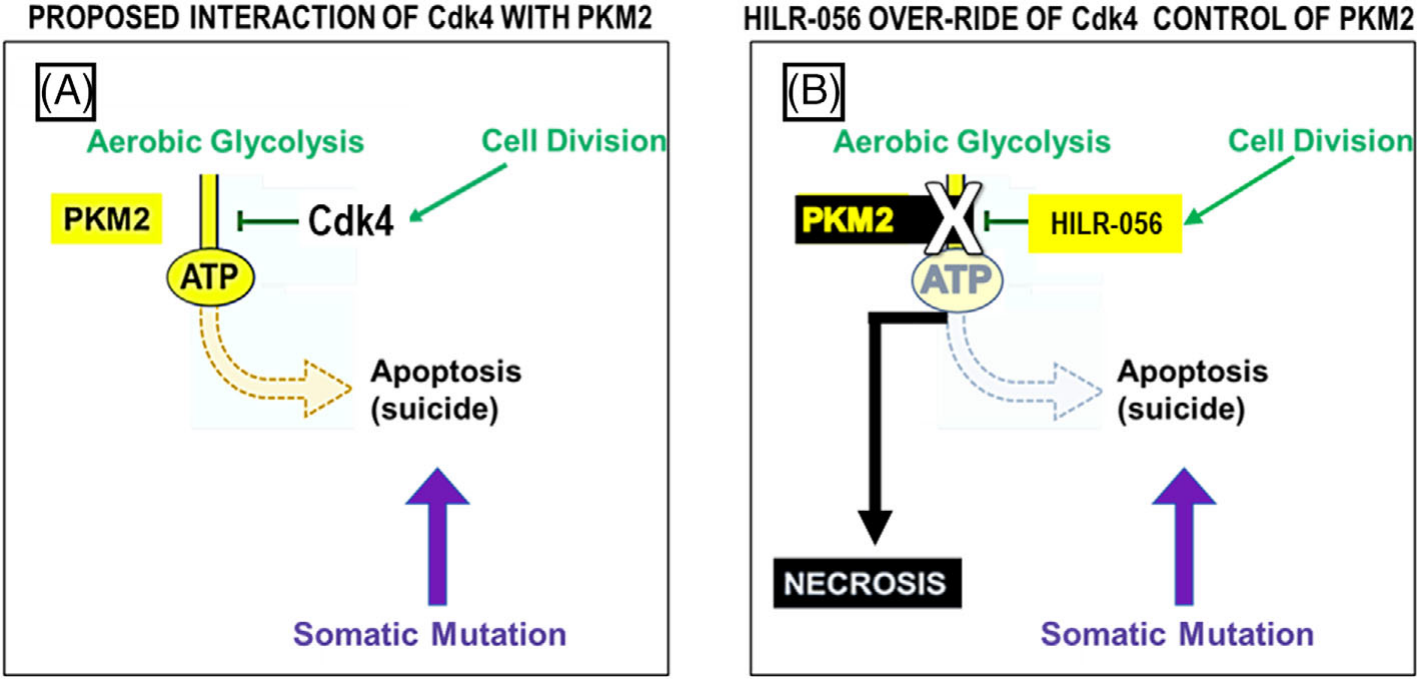
(A) A proposed Interaction of Cdk4 and PKM2 in cancer cells down‐regulates ATP production from phospho‐enol pyruvate so that there is insufficient ATP to carry out apoptosis but sufficient ATP to maintain cell survival. (B) HILR‐056 mimics the interaction of Cdk4 with PKM2 but is more potent and in higher concentration than physiological levels of PRGPRP in the NK region of external loop of Cdk4 so that there is profound depression of ATP production resulting in necrosis.

Cyclic amphiphilic peptides carrying a PRGPRP warhead or more recent congeners such as HILR‐056 in which the prolines have been substituted by very highly non‐polar unnatural amino acids with side chains such as naphthalenes and coumarins which increase their specific activity cause necrosis at 100 nM concentrations. Consistent with the hypothesis presented here these peptides may “highjack” the mechanism by which cancer cells normally avoid apoptosis (Figure 4). By virtue of their higher concentration and specific activity, they would have a greater effect of inhibition of PKM2 when they interact with SDPTEA, than happens when cancer cells avoid apoptosis, by using the NK region within the external loop in the C‐terminus of Cdk4. These novel anticancer agents kill cancer cells by necrosis rather than apoptosis. This approach introduces the possibility of a new generation of anticancer drugs that kill cancer cells by depriving them of energy rather than by attacking their replication.

## AUTHOR CONTRIBUTIONS

The ideas presented here have come solely from the author's own mind.

## FUNDING INFORMATION

This work was funded by equity release from the author's own home. There has been no charitable or commercial funding to support the experiments or ideas presented here. No external funding was received for this work.

## CONFLICT OF INTEREST STATEMENT

Hilmar M Warenius is CEO of SYNTHERIX Ltd. He is attempting to raise investment funding to take the drug he has discovered through preclinical studies and hopefully Phase I, II clinical studies, but has received no funding, investor, commercial or charitable with regard to this intention up to the present time.

## Data Availability

All data are already available in the literature or publicly filed patents. Figures 3 and 4 are from the author's own mind.
